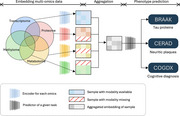# Incomplete multi‐modal learning of omics data for phenotype prediction and biomarker discovery

**DOI:** 10.1002/alz70855_100741

**Published:** 2025-12-23

**Authors:** Sungjoon Park, Kyungwook Lee, Soorin Yim, Doyeong Hwang, Kiyoung Kim, Amy R Dunn, Daniel M Gatti, Elissa J Chesler, Kristen MS O'Connell

**Affiliations:** ^1^ LG AI Research, Gangseo‐gu, Seoul, Korea, Republic of (South); ^2^ The Jackson Laboratory, Bar Harbor, ME, USA

## Abstract

**Background:**

Multi‐omics data from large‐scale consortium databases, such as the Religious Orders Study and Rush Memory and Aging Project (ROSMAP), combined with advanced AI technologies, hold significant promise for identifying biological mechanisms and biomarkers for diagnosis and treatment. However, two major challenges hinder effective multi‐omics integration: modality collapse, where prediction models overly rely on a single modality, and data incompleteness, which limits the potential of machine learning approaches to fully utilize these rich resources. To address these issues, we developed an Alzheimer's disease (AD) prediction method capable of utilizing incomplete modalities while identifying key biomarkers through feature importance analysis.

**Method:**

The proposed model is designed to ensure that each modality contributes meaningfully to phenotype prediction, even in the absence of some modalities. It comprises three modules: Encoder, Aggregator, and Predictor. Each omics data type is independently *encoded* into an embedding vector, which is then *aggregated* into a unified representation to facilitate the integration of diverse data types for AD prediction. This unified vector, along with other modality‐specific embedding vectors, is fed to a shared *predictor*. To align the heterogeneous omics embeddings, the model computes a collective loss that integrates both the unified and modality‐specific vectors, akin to main and auxiliary tasks in multi‐task learning. For missing modalities, the embedding vectors of other available modalities are amplified to compensate for the loss of information.

**Result:**

Trained on multi‐omics samples with both complete and incomplete modalities, the model achieved an accuracy of 0.890 in classifying CogDX labels, outperforming existing state‐of‐the‐art models. Ablation studies showed that all omics data contributed to the prediction, effectively preventing modality collapse. Including samples with missing modalities significantly boosted performance, emphasizing the importance of leveraging incomplete data. The model's performance was also evaluated across varying numbers of available modalities to present its practicality. Feature importance analysis identified biomarkers consistent with findings in existing literature.

**Conclusion:**

The proposed method effectively integrates multi‐omics data, even with incomplete modalities. By rediscovering biomarkers aligned with other studies, it demonstrates the potential of deep learning approaches in multi‐omics research for AD.